# Synthesis of Sulfated Sn–Zr Mesoporous Catalysts for the Selective Dehydration of Hexose‐Type Monosaccharides

**DOI:** 10.1002/open.202400480

**Published:** 2025-07-13

**Authors:** Eliana Diguilio, Horacio Falcón, Marcelo E. Domine, María Laura Martínez

**Affiliations:** ^1^ Centro de Investigación y Tecnología Química (CITeQ‐CONICET) Universidad Tecnológica Nacional ‐ Facultad Regional Córdoba Maestro M. López esq. Cruz Roja 5016 Córdoba Argentina; ^2^ Instituto de Tecnología Química (UPV ‐ CSIC) Universitat Politècnica de València. Consejo Superior de Investigaciones Científicas Avda. Los Naranjos s/n 46022 Valencia Spain; ^3^ Centro de Investigación en Nanociencia y Nanotecnología (NANOTEC) Universidad Tecnológica Nacional ‐ Facultad Regional Córdoba Maestro M. López esq. Cruz Roja 5016 Córdoba Argentina

**Keywords:** fructose dehydration, mesoporous materials, solid acids, sulfated catalysts, tin–zirconia

## Abstract

Synthesis of combined Brønsted and Lewis acid sites in SBA‐15 by Zr and Sn incorporation onto the mesoporous support, followed by a sulfation procedure to get enhanced acid catalysts for fructose‐to‐5‐hydroxymethylfurfural (HMF) selective dehydration, is here studied. Different preparation methods are used to attain the Sulfated Sn–Zr–SBA‐15 material with adequate combination of Brönsted and Lewis acidity. Samples are characterized by using different analytical and spectroscopic techniques, results indicating a correlation between the preparation procedure and the acid characteristics of the materials. The latter are evaluated in the catalytic conversion of concentrated fructose solutions (30 wt%) into HMF in a biphasic system (water + iso‐butylketone/2‐butanol). The most efficient catalyst, SO_4_/Sn–Zr–SBA‐15 (Zr/Sn molar ratio = 1.98), yields ≥ 70% HMF at a substrate conversion of ≈90% under optimal reaction conditions (150 °C for 120 min.), these results being particularly relevant when considering the higher initial fructose concentrations here used compared to those typically employed in previous reports. This catalyst exhibits a higher ratio of Bronsted/Lewis (B//L) acid sites of moderate strength and a lower density of acid sites compared to the other catalytic samples evaluated, mainly due to the presence of well‐dispersed tin and zirconium species, as well as to the enhanced acidic properties provided by sulfate groups in the material.

## Graphical abstract

The synthesized SO_4_/Sn–Zr–SBA‐15 shows high activity in fructose‐to‐5‐hydroxymethylfurfural (HMF) dehydration. Acid sites with positive synergy effect between metals and SO_4_^=^ improve catalytic behavior

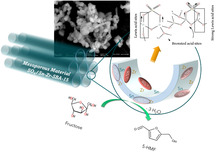

## 1 Introduction

Dehydration reactions play a crucial role in the conversion of carbohydrates into valuable platform molecules, such as furanic derivatives. Among the biomass‐derived chemicals, both furfural and 5‐hydroxymethylfurfural (HMF) have received considerable attention due to their potential for producing intermediates useful for biofuels and biochemical production.^[^ ^1^ ^]^ HMF is a versatile “platform” molecule useful for synthesizing various target chemicals,^[^ ^2^, ^3^ ^]^ which could be obtained from lignocellulosic biomass through the depolymerization, hydrolysis, isomerization, and selective dehydration of mono‐, di‐, and polysaccharides. The latest saccharide dehydration pathway involves, in general, the removal of three water molecules through acid catalysis, which can occur in either aqueous or organic media. However, the aqueous environment often promotes subsequent hydrolysis and rehydration reactions, leading to the formation of acid byproducts, such as levulinic and formic acids, as well as condensation and polymerization pathways generating soluble polymers and insoluble humins, which can decrease the selectivity to HMF.^[^ ^4^ ^]^

In this context, optimizing the production of HMF has been a focus of research for many years, with various methods developed to achieve high yields from fructose as the starting reactant. Cope et al. initially proposed a water‐organic solvent biphasic system for the continuous extraction, stabilization, and isolation of HMF. Effective organic solvents identified include methyl isobutyl ketone (MIBK), n‐hexanol, diethyl ether, and diethyl ketone, with MIBK being favored due to its commercial availability, low cost, and favorable distribution coefficient of 0.79.^[^ ^5^ ^]^ Meanwhile, Román‐Leshkov et al. investigated a two‐phase system for fructose dehydration using an acid catalyst in the aqueous phase, with dimethyl sulfoxide (DMSO) and/or poly(1‐vinyl‐2‐pyrrolidinone) added to suppress undesired side reactions. The HMF product is continuously extracted into an organic phase (MIBK) modified with 2‐butanol to enhance HMF production.^[^ ^6^ ^]^

In the last years, various acid catalysts have been employed for the conversion of saccharides to HMF, including homogeneous catalysts such as mineral acids, organic acids, ionic liquids, and Lewis acids (e.g., metallic chlorides), as well as heterogeneous catalysts like metallic oxides, phosphates, heteropoly acid compounds, zeolites, mesoporous solids, and ion exchange resins.^[^ ^7^, ^8^ ^]^ Nevertheless, one of the main drawbacks encountered when most of the solid acid catalysts are used consisted of the rapid deactivation of acid sites due to carbonaceous compound deposition (i.e., humins), this leading to low HMF yields during the process. The formation of these humin‐type oligomeric compounds is usually related to consecutive acid‐catalyzed reactions, their occurrence being favored when strong acid sites are relatively close to one another in the solid catalyst surface. In this context, the incorporation of well‐ and homogeneously dispersed strong acid sites onto high surface area supports, particularly mesoporous materials, has garnered significant attention for sugar‐to‐furan transformations, because they could reduce or even overcome humin production, for example, by enlarging the distances between strong acid sites in solid catalysts.

Silicon mesoporous materials were first discovered by researchers from Mobil Corporation,^[^ ^9^ ^]^ utilizing structure‐directing agents, known as templates or surfactants, which facilitate the formation of the porous system. These materials were classified as the M41S family, with MCM‐41 being the flagship due to its two‐dimensional porous structure characterized by hexagonal pores and high surface areas. In 1998, Zhao and colleagues^[^ ^10^ ^]^ reported the synthesis of the SBA‐s family, further advancing the field of mesoporous materials. Among these, SBA‐15 has attracted considerable interest due to its enhanced properties compared to MCM‐41, including increased wall thickness and interconnected microporous channels, which contribute to greater hydrothermal stability. According to the international union of pure and applied chemistry,^[^ ^11^ ^]^ SBA‐15 is classified as a mesoporous material with pore diameters ranging from 2 to 50 nm, and its amorphous structure is another significant characteristic.

Due to their neutral siliceous form, SBA‐15 mesoporous materials can be modified and functionalized in various ways, including the addition of sulfonic groups, amines, and the immobilization of enzymes, as well as serving as support for metals that provide acidity and/or nother functionalities.^[^ ^12^ ^]^ These functionalization methods enable a wide range of applications for the resulting materials, such as controlled drug release,^[^ ^13^ ^]^ adsorption,^[^ ^14^ ^]^ and catalysis.^[^ ^15^, ^16^, ^17^ ^]^

Recent studies have focused on the properties of SiO_2_–ZrO_2_ mixed oxides,^[^ ^18^, ^19^, ^20^ ^]^ which exhibit favorable physicochemical characteristics, catalytic activity, and specific surface acidity. These materials have diverse applications, including superacid solids and heterogeneous acid‐type catalysis. Investigations into mixtures of zirconium and silicon oxides in contact with sulfuric acid solutions have demonstrated the formation of strong acid sites, which have been effectively applied in n‐hexane isomerization reactions.^[^ ^21^ ^]^

The ongoing pursuit of new functionalities in siliceous SBA‐15 materials has led to the incorporation of tin. Tin, being in the same group as silicon in the periodic table, shares similar oxidation states, facilitating its introduction into the silicon–oxygen network. When tin species are integrated into mesoporous matrices, they can interact with surface hydroxyl groups or replace silicon (Si^4^^+^), resulting in Sn—O—Si bonds. This incorporation is relatively straightforward, as it does not disrupt charge balance. Consequently, tin‐supported materials on mesoporous substrates have found extensive use in catalysis.^[^ ^22^, ^23^, ^24^ ^]^ The addition of tin to mesoporous materials generates efficient Lewis acid sites capable of catalyzing specific reactions where Brønsted acid sites may be less effective or unsuitable. For instance, Zhang and coworkers^[^ ^25^ ^]^ synthesized tin‐functionalized SBA‐15 with sulfonic acid, applying the catalytic material in the esterification of oleic acid to produce biodiesel, thereby highlighting the importance of the specific acidity of the modified SBA‐15 material for the reaction.

Building on this foundation, the synthesis of novel acid‐functionalized SBA‐15 mesoporous materials remains a topic of great interest for both academic and industrial researchers. These materials can be utilized for various applications aimed at addressing the increasing demand for reliable, renewable, and sustainable energy sources, particularly through catalytic transformations of lignocellulosic biomass and its derivatives.

In this sense, extensive studies have been conducted on sulfated metal oxides as superacid catalysts.^[^ ^26^, ^27^ ^]^ A prominent example of this type of superacid is sulfated zirconia (SO_4_/ZrO_2_), which has demonstrated high catalytic activity in a variety of chemical reactions. Aguzin and coworkers synthesized sulfated zirconia on SBA‐15 mesoporous material using different zirconium molar ratios (Si/Zr = 10, 20, 30) to obtain diethyl succinate (DES) via the catalytic esterification of succinic acid with ethanol. The results revealed that the catalytic activity of the developed catalyst was significantly superior to that obtained using Amberlyst 36.^[^ ^28^ ^]^ Moreover, sulfated tin oxide (SO_4_/SnO_2_) has emerged as a candidate exhibiting superior acidity on its surface, with reported acid strength exceeding that of SO_4_/ZrO_2_.^[^ ^29^ ^]^ E. Tututi‐Ríos et al.^[^ ^30^ ^]^ demonstrated that Sn‐SBA‐15 modified with ‐PrSO_3_H (propylsulfonic) groups possesses enhanced acidic properties. Tin species primarily introduce Lewis acid sites, which are essential for catalytic processes that require the activation of molecules with electron pairs. The incorporation of sulfonate groups was crucial for generating Brønsted acid sites, which increased the acidity of the material (with a Brønsted/Lewis ratio of ≈4:1). Thus, the combination of Sn and ‐PrSO_3_H enabled the catalyst to exhibit both Lewis and Brønsted acidities, thereby markedly improving its activity in the esterification reactions of fatty acids with methanol.

Following this line of thinking and regarding the transformation of sugars to furanic compounds, sulfated zirconia remains one of the most recognized superacid solids for the synthesis of HMF and is considered highly attractive for industrial chemical applications.^[^ ^31^ ^]^ Its Brønsted and Lewis acid sites are key to promoting fructose dehydration to HMF. Nonetheless, its catalytic performance is influenced by factors such as surface area, porosity, and thermal stability,^[^ ^32^ ^]^ which have limited its scalability for industrial applications.

In contrast, SBA‐15 mesoporous material offers several intrinsic properties that make it ideal for catalysis—such as thermal stability, high specific surface area, a highly ordered mesostructure, and amenability to surface modification. To improve both its thermal and catalytic behavior, Madarász et al.^[^ ^33^ ^]^ incorporated sulfated zirconia and metallic magnesium into SBA‐15 pores, applying the modified material successfully in fructose dehydration. Moreover, Zhang et al. further developed SBA‐15‐based bifunctional catalysts by incorporating monolayers of sulfated zirconia along with acid–base groups, enabling the conversion of cellulose into 5‐HMF.^[^ ^34^ ^]^ In addition, other studies have confirmed the effectiveness of introducing sulfonic acid groups into SBA‐15 for fructose dehydration, with excellent catalytic outcomes reported even under harsh conditions and in the presence of costly reagents like ionic liquids.^[^ ^35^, ^36^, ^37^ ^]^

Finally, the introduction of tin (Sn) into mesoporous supports has proven to be an effective strategy for generating Lewis acid sites useful for 5‐HMF synthesis from sugars. Thus, Lorenti et al.^[^ ^38^ ^]^ evaluated Sn‐loaded SBA‐15 for glucose conversion and achieved HMF yields up to 57%. In general, SBA‐15 mesoporous material provides high applicability in catalysis due to its thick pore walls, which enhance thermomechanical stability.^[^ ^39^ ^]^ In this sense, incorporating Sn into mesoporous SBA‐15 is considered a beneficial strategy to enhance catalytic performance in the production of 5‐HMF.

With all the above‐mentioned in mind, we propose in this research work the synthesis of combined Brønsted and Lewis acid sites in SBA‐15 mesoporous material by Zr and Sn incorporation onto the mesoporous support, followed by a sulfation procedure. As far as we know, this is the first study on a strong acidic material obtained from the interaction between two transition metals (tin and zirconium) combined with sulfated species onto SBA‐15. Different preparation methods are used to attain the sulfated Sn–Zr–SBA‐15 with adequate combination of Brönsted and Lewis acidities. The obtained solids were characterized using different analytical and spectroscopic techniques and then tested as catalysts in fructose dehydration reaction for selective HMF production. The main objective of this research is to analyze the interaction between tin and zirconium with sulfate‐type species in the creation of strong acidic materials, as well as their influence on the attained HMF yields during the fructose dehydration reaction.

## 2 Results and Discussion

### 2.1 Structural and Textural Analysis

The main textural properties of prepared catalysts and their SBA‐15 mesoporous precursor were analyzed by N_2_ adsorption–desorption isotherms, and the results are exposed in **Table** **1**. The SBA‐15 matrix presented a very high surface area (648 m^2^ g^−1^), an average pore diameter of 6.8 nm, and a pore volume of 0.84 mL g^−1^. The incorporation of Zr produced a decrease in the surface area and pore volume, while the simultaneous incorporation of Zr and Sn reduced the surface area, as well as the pore size and volume. The addition of zirconium and subsequent sulfation in SO_4_/Zr–SBA‐15 sample also produced a decrease in the surface area, pore size, and volume. Moreover, the tin incorporation and sulfation processes over the Zr–SBA‐15 sample resulted in a significant decrease in surface areas while only moderately affecting pore size and volume. The decrease in the surface area was similar for both tin incorporation methods (before or after sulfation).

**Table 1:** Main textural properties of calcined SBA‐15‐based mesoporous materials.

Sample	Surface area [m^2^ g^−1^]	Average pore size [nm]	Pore volume [cm^3^ g^−1^]
SBA‐15	774	6.8	0.980
Zr–SBA‐15	588	6.7	0.732
Sn–Zr–SBA‐15	513	6.2	0.720
SO_4_/Zr–SBA‐15	358	5.0	0.685
SO_4_/Sn–Zr–SBA‐15	252	4.9	0.560
Sn–SO_4_/Zr–SBA‐15	240	4.5	0.550

For a better understanding of the data presented in Table 1, Figure S1, Supporting Information has been depicted (see Supporting Information). Figure S1, Supporting Information shows that the incorporation of zirconium and tin does not lead to a significant decrease in the surface area, pore size, or pore volume of the support, suggesting that the metal oxides have been introduced with adequate dispersion both on the surface and within the nanometer‐sized pores. In contrast, the sulfation treatment applied to the materials results in a considerable loss of textural properties (surface area, pore size, and pore volume). This effect may be attributed to the interaction of the sulfate anion with the surface of the support and the metal oxides, potentially causing partial pore blockage, as illustrated graphically, as well as to the structural loss caused by successive postsynthesis treatments.

**Figure 1:**
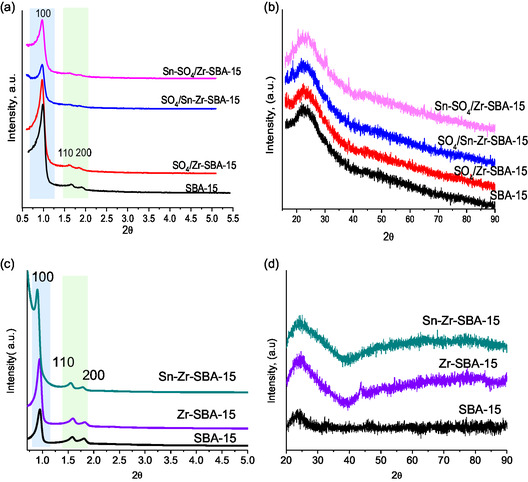
Low a,c) and wide b,d)‐angle XRD patterns of metallic and sulfated SBA‐15 mesoporous materials.

**Figure** **1**a,c shows diffraction patterns of the calcined pure SBA‐15, metal–SBA‐15, and sulfated‐metal–SBA‐15 samples in the low‐angle region between 0.5 and 7.0 degrees of 2*θ*. In all samples, porous ordering can be observed, exhibiting three well‐resolved diffraction peaks with Miller indices (100), (110), and (200), which are associated with porous hexagonal structures with a p6mm symmetry. SBA‐15 as synthesized possesses an intense peak at 0.96 of 2*θ*, corresponding to the (100) plane and two small peaks between 1.58 and 1.80º for (110) and (200), respectively, in concordance with the hexagonal typical pore arrangement.^[^ ^10^ ^]^

The X‐ray diffraction patterns show that after performing the functionalization, all catalysts retain the SBA‐15 mesoporous material characteristic peaks. The presence of these distinctive peaks states that all materials conserve their pore array despite treatments. Furthermore, it can be observed that as postsynthesis treatments are applied to the materials, there is a decrease in the peak intensities, indicating a slight loss of the mesoporosity. Additionally, **Table** **2** presents a summary of the data obtained from structural and textural analyses of the prepared SBA‐15 mesoporous materials. It can be observed that as different functionalizations are carried out, the interplanar distance (d_0_) increases from 92.9 to 97.9 (**Å**), leading to an increase in the lattice parameter. This phenomenon in lattice parameters can be attributed to the incorporation of metals (Zr–Sn) into the Si–O network; they possess larger ionic diameters relative to silicon. This is consistent with previous literature reports.^[^ ^40^, ^41^ ^]^ Similarly, there is an increase in the wall thickness of the samples, which is calculated by means of the equation: *t* = a_0_ – average pore size. This suggests that, while there is a decrease in the surface area with successive impregnations and sulfations, the pore diameter proportionally decreases. This leads us to conclude that the metal and sulfate ions could be localized themselves within the pores of the synthesized materials.

**Table 2:** Results of structural and textural analysis of calcined SBA‐15 mesoporous samples.

Sample	2 thetas	*d*_0_ [Å]	*a*_0_ [Å]	Wall thickness [Å]a)
SBA‐15	0.96	92.9	106.2	38.2
Zr–SBA‐15	0.94	93.7	108.2	41.2
Zr–Sn–SBA‐15	0.90	97.5	112.6	50.6
SO_4_/Zr–SBA‐15	0.92	96.1	110.9	59.9
Sn–SO_4_/Zr–SBA‐15	0.90	97.4	112.5	67.5
SO_4_/Sn–Zr–SBA‐15	0.89	97.9	113.1	64.1

a) Calculated as: (a_0_‐Average pore size).

In addition, Figure 1b,d shows the wide‐angle X‐ray diffraction patterns, which display only a broad peak at around 2*θ* = 23°, which is typical of the amorphous structure of the silica walls of SBA‐15. The small peak close to 2*θ* = 30.3° for Sn–SO_4_/Zr‐based sample is attributed to amorphous ZrO_2_.^[^ ^42^ ^]^ Overall, X‐ray diffractograms suggest that the incorporation of metallic oxides and the sulfation treatment do not generate consistently large clusters of zirconium or tin oxides.

In Figure 1d, the catalysts do not exhibit any distinct diffraction peaks; however, a noticeable increase in the baseline is observed in the 2*θ* range of 40–90°. This phenomenon may be attributed to the high dispersion of zirconium and tin oxides, which are likely present as nanoclusters distributed both on the surface and within the pores of the support. Such a dispersion could lead to the absence of characteristic diffraction peaks of the incorporated metal oxides due to the confinement effect associated with the size of the mesoporous structure,^[^ ^43^ ^]^ although some structural changes induced by the incorporation of tin (Sn) into the zirconium (Zr) matrix could not be ruled out.

### 2.2 Acidity Measurements

**Figure 2:**
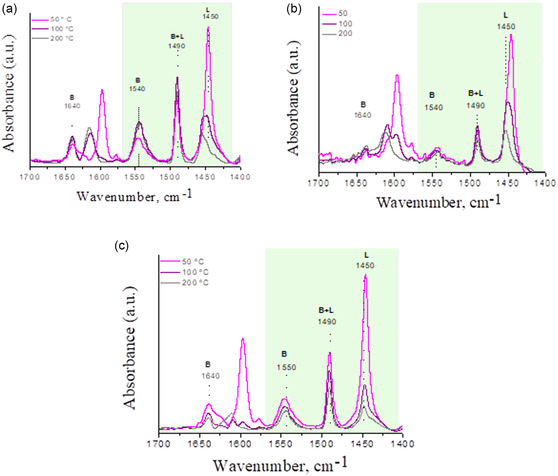
Desorbed Py‐FTIR spectra of a) SO_4_/Sn–Zr–SBA‐15, b) Sn–SO_4_/Zr–SBA‐15, and c) SO_4_/Zr–SBA‐15 materials at 50, 100, and 200 °C, respectively.

The acid properties of the prepared materials were analyzed by FTIR with pyridine (Py) adsorption and subsequent desorption at different temperatures. The Py‐FTIR spectra (only sulfated materials), shown in **Figure** **2**, exhibit the presence of adsorbed molecules of pyridine at both Lewis and Brønsted acid sites. Thus, bands at 1446–1450 and 1540 cm^−1^ were observed and assigned to pyridine adsorbed in the OH groups corresponding to Lewis and Brønsted acid sites, respectively. The desorption spectra of Py at different temperatures show a marked decrease in the pyridine adsorption signal at 1446 cm^−1^, corresponding to surface hydroxyl groups bonded to H bridges with pyridine. This fact indicates that in sulfated Sn and/or Zr mesoporous materials, the surface OH groups are relatively weaker in terms of acid strength.^[^ ^44^ ^]^ On the other hand, the reduction of the signal corresponding to adsorbed pyridine at Brønsted acid sites at different temperatures is less pronounced, indicating a stronger acidity due to sulfate ions and heteroatoms incorporated into the material. Lewis's acidity also appears to exhibit some stability during thermal treatments. The evaluation of Py‐FTIR band spectra at 1575 cm^−1^ shows low‐intensity peaks of adsorbed pyridine in the samples after desorption treatment at 50 °C. The SO_4_/Zr/SBA‐15 material exhibits slight Lewis and Brønsted acidities, as low‐intensity signals are detected for all desorption temperatures, whereas Sn–SO_4_/Zr–SBA‐15 exhibits slightly stronger signals, especially those corresponding to Lewis acidity. This is probably due to the incorporation of Sn, favored by sulfate ions anchored on the surface, which provide characteristic metallic acidity. The SO_4_/Sn–Zr–SBA‐15 material exhibits the highest intensity in the bands corresponding to Lewis and Brønsted acid sites.^[^ ^30^ ^]^

On one hand, the synthesis of the SO_4_/Sn–Zr–SBA‐15 material was made through coimpregnation of Sn and Zr; this probably gives a higher dispersion of both metals onto the surface of the SBA‐15 mesoporous material. On the other hand, materials after sulfation procedure (treated with H_2_SO_4_) showed more amounts of Bronsted acid sites, this probably conducting a better acid catalytic activity. However, there are differences in the amount of Lewis and Bronsted acid sites generated in different sulfated samples. **Table** **3** shows that SO_4_/Zr–SBA‐15 has a high quantity of Lewis and Bronsted sites. It can be inferred that sulfate species are bonded on tetrahedral zirconium and surface silanol groups. When this material (SO_4_/Zr–SBA‐15) is impregnated with tin to generate the Sn–SO_4_/Zr–SBA‐15 sample, it is observed that these sites decrease abruptly, possibly due to tin oxides covering or blocking the acid sites previously formed. However, in the SO_4_/Sn/Zr–SBA‐15 material, a lower number of Lewis and Bronsted acid sites was determined. It could be hypothesized that the treatment with metal precursors on the surface of SBA‐15 (before sulfation treatment) generates surface bonds of metals with silanol groups, incorporating them in a tetrahedral manner in the SBA‐15 structure and leaving fewer OH groups for the sulfate ions to anchor to. This could result in a lower number of both Lewis and Bronsted acid sites in the final solid. Moreover, the ratios of Brønsted/Lewis's acid sites (B/L) were calculated from Py‐FTIR spectra obtained at different desorption temperatures (see Table S1, Supporting Information), and those attained for desorption at 200 °C are shown in Table 3. It was found that in all the cases, the B/L ratio increases at higher outgassing temperature (see Table S1, Supporting Information). The materials combining the presence of tin and sulfate species showed a higher B/L ratio than the analogous samples without this metal (for example, SO_4_/Zr–SBA‐15), and the increase in the B/L ratio with outgassing temperature depended on the way that tin metal was incorporated (see Table S2, Supporting Information). The sample obtained by adding tin before sulfation (SO_4_/Sn–Zr–SBA‐15) showed a moderate increase in the B/L ratio with temperature, while the sample obtained by tin incorporation after sulfation (Sn–SO_4_/Zr–SBA‐15) showed a higher slope in the B/L relationship with the outgassing temperature (see Table S2, Supporting Information). From results of Table 3, it becomes clear that the SO_4_/Zr–SBA‐15 sample possesses a higher total acidity with predominant Lewis acid sites, this resulting in the lower B/L ratio and the highest density of acid sites (calculated as total acid sites divided by the surface area, mmol Py g m^−2^). Remarkably, the combined presence of tin and sulfate species in materials provides a B/L ratio close to one, while the coimpregnation of tin and zirconium on the SBA‐15 before sulfation gives the highest B/L ratio accompanied by the lowest total acid sites density. The latter behavior of SO_4_/Sn–Zr–SBA‐15 is probably due to the high metal dispersion achieved with metal coimpregnation procedure.

**Table 3:** Brønsted and Lewis acid sites determined by FTIR of pyridine adsorption and ulterior desorption at 200 °C.

Catalyst	Lewis acid sites [mmol Py g^−1^]	Brønsted acid sites [mmol Py g^−1^]	Total acid sites [mmol Py g^−1^]	B/L ratio	Surface Area [m^2^ g^−1^]	Density of acid sites [mmol Py g m^−2^]
Zr–SBA‐15	48.72	28.45	77.18	0.58	588	0.131
Sn–Zr–SBA‐15	56.03	28.66	84.68	0.51	513	0.165
SO_4_/Zr–SBA‐15	187.24	79.47	266.71	0.42	358	0.745
Sn–SO_4_/Zr–SBA‐15	49.41	45.90	95.31	0.93	242	0.394
SO_4_/Sn–Zr–SBA‐15a)	15.27	16.47	31.74	1.08	252	0.126

a) Calculated by desorption at 400 °C.

The incorporation of tin into the Zr–SBA‐15 material, followed by subsequent sulfation (SO_4_/Sn–Zr–SBA‐15), shows a linear increase in the number of Lewis acid sites. This suggests that both Sn incorporation and the addition of SO_4_^2^^−^ groups contribute moderately to the generation of Lewis acidity. When analyzing the number of Brønsted acid sites, it remains nearly constant between the Zr–SBA‐15 and Sn–Zr–SBA‐15 materials. However, an exponential increase is observed in the SO_4_/Sn–Zr–SBA‐15 catalyst (see Figure S3, Supporting Information). This indicates that sulfate groups interact with both Sn^4^^+^ and Zr^4^^+^ species. The presence of SO_4_^2^^−^ groups enhance the Brønsted acidity of the catalyst, and the Zr/Sn ratio may influence the way these acidic groups anchor onto the support.

The Sn and Zr atoms have a high affinity for oxygen groups, allowing sulfate groups to anchor to the surface of these metals through electrostatic interactions and covalent bonds. Under sulfatation conditions, the SO_4_^2^^−^ groups coordinate to the metal centers by forming M—O—S bonds, where M can be either Sn or Zr. This anchoring alters the electronic distribution around the metals, modifying the acidic properties of the material. Zr, in the absence of sulfate groups, tends to primarily generate Lewis acid sites, as its metal centers directly coordinate with electron‐donating molecules. However, when sulfate groups anchor to Zr, they act as Brønsted acids, since the SO_4_^2^^−^ groups can donate protons (H^+^) in aqueous solutions, creating Brønsted acid sites on the catalyst surface.

Sn also generates Lewis sites when in a nonsulfated form, but the introduction of sulfate groups onto Sn centers modifies their chemical environment, reducing the metal's electron density. This results in a decrease in Lewis sites in favor of the formation of Brønsted sites, as the SO_4_^2^^−^ groups, when anchored to the Sn surface, can generate protons that act as Brønsted acids. Lewis acid sites are primarily associated with metal centers that act as electron acceptors, coordinating with electron‐donating molecules. In the case of Sn and Zr, these metals form Lewis sites by attracting ligands with unshared electron pairs. However, when sulfate groups are anchored onto these metals, the available electrons from the metal are redistributed due to the sulfate's strong affinity for the metal atoms. This diminishes the metal center's ability to act as a Lewis acid site, as part of its electron density is committed to maintaining the bond with the sulfate groups.

**Figure 3:**
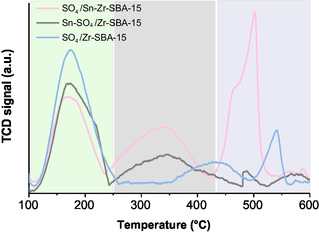
NH_3_‐TPD profiles of SO_4_/Zr–SBA‐15, Sn–SO_4_/Zr–SBA‐15, and SO_4_/Sn–Zr–SBA‐15 samples.

Ammonia temperature‐programmed desorption (NH_3_‐TPD) experiments were also performed to study the acid sites of the three sulfated materials prepared in this work. As shown in **Figure** **3**, the desorption curve of each sulfated sample exhibited three temperature regions, 110–250, 250–450, and 450–600 °C, corresponding to weak, moderate, and strong acid sites, respectively. The curves were generated exclusively due to desorbed ammonia contributions as the sulfate groups remain anchored to zirconia‐containing materials for temperatures up to 650 °C,^[^ ^45^ ^]^ and no further peaks were observed for both tin‐containing samples. The addition of tin over sulfated zirconia (Sn–SO_4_/Zr–SBA‐15) caused the weak acidity region curve to become broader, corresponding to the Lewis acidity gain determined by FTIR of adsorbed pyridine. However, for the sample where the sulfation process was performed after Sn incorporation (SO_4_/Sn–Zr–SBA‐15), the intensity of this signal was reduced. This was expected, as the sulfate groups grafted to Zr and Sn sites blocked the access of the NH_3_ to these Lewis acid sites. In the moderate acidity region, the tin‐containing materials exhibit an abundance of acid sites with a desorption temperature close to 320–340 °C, indicating the presence of Lewis's acid sites with moderate strength. These moderate strength acid sites or at least the corresponding NH_3_ desorption bands are not observed in the case of SO_4_/Zr–SBA‐15 sample. The strong‐acidity region, usually ascribed to Brønsted acid sites, behaved differently in each case. For SO_4_/Zr–SBA‐15, the curve showed a maximum intensity signal at ≈540 °C. With tin incorporation in this sample (Sn–SO_4_/Zr–SBA‐15), this peak split into two weak signals with maxima at 490 and 575 °C, respectively. On the contrary, when the sulfation process took place after Sn incorporation (SO_4_/Sn–Zr–SBA‐15 sample), a much more intense and broader peak was observed shifted at lower temperatures. This signal was assigned to strong Brønsted acid sites, described by the contribution of two main signals, maximized at 470 and 500 °C NH_3_ desorption temperatures, respectively. This fact supports the hypothesis that sulfation occurs in both zirconia and tin Lewis's acid sites on SBA‐15 material, and it is also in agreement with previously reported sulfation treatments over tin oxides.^[^ ^46^, ^47^ ^]^

### 2.3 Catalytic Performance Studies

#### 2.3.1 Screening of Catalysts

The catalytic activity of the synthesized solids was evaluated in the selective dehydration of fructose to HMF by using an aqueous/organic solvent biphasic system. In the case of the organic phase, the solvent mixture composed by MIBK/2‐butanol (7:3 in weight) with higher HMF extraction efficiency compared to that of MIBK alone (*K*_H_ = 1.57 for H_2_O/MIBK‐2‐butanol vs. *K*_H_ = 1.09 for H_2_O/MIBK) was employed. Thus, the higher the K_H_, the greater HMF concentration in the organic phase relative to the aqueous phase, which ensures a better HMF extraction to the organic phase, protecting it from rehydration and polymerization reactions that occur in an acidic aqueous medium. This mixture of organic solvents (MIBK + 2‐butanol) possesses the ability to solubilize substantial quantities of HMF without leading to its decomposition. When compared to systems that utilize only MIBK, the inclusion of 2‐butanol significantly improves the efficiency of extraction and mitigates the necessity for additional high‐boiling‐point solvents, which often entail more expensive and energy‐intensive separation processes.^[^ ^6^, ^48^ ^]^ On the contrary, in studies employing DMSO as a solvent, high fructose conversions are achievable by reducing the concentration of HMF in the reaction medium and, consequently, minimizing its rehydration and the formation of humins.^[^ ^49^ ^]^ Nevertheless, the process encounters significant challenges in the separation of HMF due to DMSO's strong affinity for water, necessitating extensive distillation processes to recover HMF.

**Figure 4:**
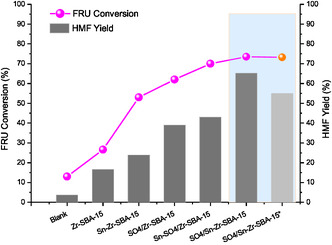
Conversion of fructose and HMF yield for dehydration reactions performed in aqueous/organic MIBK‐2 butanol (7:3) biphasic solvent system (FO/FA: 70/30), at 150 °C during 120 min, employing 50 mg of catalyst (*data obtained at a 60 min reaction time).

As shown in **Figure** **4**, a series of SBA‐15‐based catalysts prepared in this work have been evaluated in fructose dehydration to obtain HMF in an aqueous/organic biphasic system. It was observed that catalysts containing sulfate groups reached better results regarding fructose conversion and HMF yield than those catalysts without sulfate species, such as Zr–SBA‐15 and Zr–Sn–SBA‐15, respectively. In addition, catalysts combining the presence of tin and sulfate species in the Zr–SBA‐15 structure presented much better behavior than the above‐mentioned metal modified (Zr–SBA‐15 and Sn–Zr–SBA‐15) and Zr‐sulfated (SO_4_/Zr–SBA‐15) mesoporous materials. In fact, the higher catalytic activities were attained, with the catalysts having both Zr and Sn metals, as well as the presence of sulfate groups. Moreover, some differences were observed between the catalysts in which tin incorporation was made before or after sulfation procedure. Thus, the SO_4_/Sn–Zr–SBA‐15 catalyst was more active than Sn–SO_4_/Zr–SBA‐15 by achieving higher fructose conversion and HMF yield at 60 min of reaction than the latter after 120 min and employing a concentrated fructose aqueous solution (30 wt%).

Results of catalytic screening demonstrated that the SO_4_/Sn–Zr–SBA‐15 mesoporous material was the most efficient catalyst for fructose dehydration to HMF, yielding 65.2% of HMF (87.1% selectivity to HMF) at a 73.1% fructose conversion by working at 150 °C for 120 min. These results are very promising taking into account the high concentration of fructose (30 wt% in aqueous solution) employed in these tests, compared to the usual values published in the bibliography (≤5 wt%) by using mesoporous materials and a less environmentally friendly solvent, such as DMSO.^[^ ^50^ ^]^ These catalytic results indicate that the contribution of both zirconium and tin active sites, as well as the sulfated metallic species, is necessary for reaching higher fructose conversion and HMF selectivity. However, the order of these treatments (metal incorporation and sulfation procedure) is also an important factor, and SO_4_/Sn–Zr–SBA‐15 catalyst showed higher yield to HMF than that of Sn–SO_4_/Zr–SBA‐15, in which the sulfation was made before tin addition. Apparently, SO_4_/Sn–Zr–SBA‐15 catalyst has been sulfated in both zirconia and tin Lewis's acid sites, and this fact would affect the acid properties of the materials. **Table** **4** reveals the amount of Py desorbed at different temperatures on the SO_4_/Sn–Zr–SBA‐15 catalyst compared to that for the nonsulfated material (Sn–Zr–SBA‐15). The desorption data at 100 °C shows that the nonsulfated material has a higher amount of Lewis sites (≈60%) and a lower amount of Brönsted acid sites (≈37%). Although the total acid sites are higher in the sulfated sample (SO_4_/Sn–Zr–SBA‐15), therefore, an increase in the number of Brönsted sites leads to better results in fructose conversion and yields toward HMF. Data of Py desorption at 300 °C also show that the sulfated material possesses ≈50% more total acid sites compared to Sn–Zr–SBA‐15 from data; however, the same does not occur at 400 °C. This indicates that the sulfated material possesses Brönsted and Lewis acid sites of moderate or medium strength.

**Table 4:** Data of Py adsorption and desorption at different temperatures over SO_4_/Sn–Zr–SBA‐15 and Sn–Zr–SBA‐15 samples measured by FT‐IR.

Catalyst	T [°C]	L [μmol Py/gr]	B [μmol Py/gr]	B/L	Total acid sites
SO_4_/Sn–Zr–SBA‐15 (Zr/Sn = 1.98)	100	88.95	137.46	1.55	226.41
	300	40.15	55.27	1.38	95.42
	400	15.27	16.47	1.08	31.74
Sn–Zr–SBA‐15 (Zr/Sn = 1.98)	100	142.79	50.96	0.36	193.75
	300	48.11	18.68	0.39	66.79
	400	28.80	10.43	0.36	39.23

**Figure 5:**
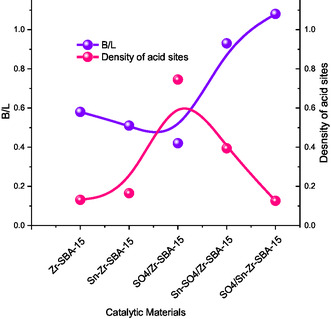
B/L acid sites ratio versus density of total acid sites for different catalysts tested (from data of Table 3).

More importantly, it seems there exists an optimum ratio of Brønsted/Lewis (B/L) acid sites, allowing attaining high catalytic activity during fructose dehydration to HMF. In fact, according to **Figure** **5**, the sample with a higher B/L ratio (SO_4_/Sn–Zr–SBA‐15) and minor density of total acid sites compared with the rest of catalytic materials, it was more active than those presenting lower values of B/L ratio and superior density of total acid sites. The results obtained are consistent with previously published findings,^[^ ^51^ ^]^ which indicate that the presence of both Brønsted and Lewis acid sites is essential for a highly active and selective catalyst in the dehydration of fructose to HMF. From our results, the optimal B/L ratio is observed in the case of the SO_4_/Sn–Zr–SBA‐15 catalyst.

Analysis of these data clearly indicate that the HMF formed during fructose dehydration strongly depends on the presence of Brönsted (B) acid sites, as well as on the B/L ratio in the catalysts. This trend correlates with the presence of sulfate species in the catalysts, which increases the catalytic activity (fructose conversion) and, simultaneously, the HMF production. Nevertheless, the extent of HMF formation also depends on the suppression of secondary and mainly consecutive reactions occurring once HMF is formed, this leading to a decrease in the HMF selectivity and the final HMF achieved. As was mentioned in the Introduction section, the proximity of strong acid sites in the catalyst could favor the occurrence of these secondary and consecutive nondesired reactions leading to the formation of side products (mainly humins). In these cases, enlarging the distances between strong acid sites in solid catalysts, this meaning decreasing the density of acid sites in the surface of the solid catalyst, allows overcoming this issue (furanic compound oligomerization).^[^ ^52^ ^]^ Therefore, the catalytic activity and mainly the HMF yield are increased when strong Brönsted acid sites are present, with a high B/L ratio and, simultaneously, minimizing the density of acid sites, which correlates with data obtained for our catalysts and is depicted in Figure 5.

#### 2.3.2 Evaluation of Reaction Parameters

Different reaction parameters have been evaluated in the fructose to HMF dehydration, by employing the SO_4_/Sn–Zr–SBA‐15 optimum catalyst and a fructose aqueous solution of 30 wt%. The catalytic performance attained at (a) different temperatures, (b) different reaction times, and (c) different amounts of catalysts is provided in Figure S2, Supporting Information. Figure S2a, Supporting Information shows the profiles of fructose conversion and HMF yield with reaction temperature ranging from 120 to 150 °C. It seems that fructose conversion increases with temperature, from 16.5% at 120 °C to 47.5% at 150 °C (for 60 min of reaction), while HMF yield also increases with temperature up to 140 °C and then decreasing slightly at 150 °C. This is probably due to the formation of polymerization products as humins, which are favored at higher temperatures and extended reaction times.^[^ ^45^ ^]^ Likewise, based on these experimental results, further studies will be continued at 150 °C because of high sugar conversion achieved and some effects of the reaction conditions can be better distinguished.

On the other hand, reaction time is a key factor in the dehydration of fructose to HMF, since at prolonged times, parallel reactions are possible, such as HMF rehydration to produce formic and levulinic acids, as well as etherification, dehydration, and condensation reactions of carbohydrates, generating the so‐called humin compounds. The fructose conversion profiles obtained from catalytic tests performed at reaction times ranging from 30 to 150 min showed an increase in substrate consumption with the time, reaching 73.1% at 120 min and c.a. 80% at 150 min, respectively (Figure S2b, Supporting Information). Meanwhile, the HMF yield increased to 45.2% at 120 min but decreasing at longer reaction times to 25.2%. This effect can be attributed to undesired secondary reactions, which are favored at longer reaction times, resulting in a significant decrease in HMF selectivity. For this reason, the optimal reaction time was established at 120 min, where the HMF yield reached the highest.

Lastly, the substrate/catalyst mass ratio was evaluated varying the amount of catalyst, employing 25, 50, and 75 mg under the optimal reaction conditions previously selected (at 150 °C for 120 min.). As can be seen in Figure S2c, Supporting Information, the fructose conversion increased with the catalyst amount from 44.6% (25 mg of catalyst) up to 78.2% with 75 mg of catalyst. However, the HMF yield showed a different behavior by increasing with the amount of catalyst from 25 to 50 mg (40.6 to 65.2%) and then decreasing to 50.1%. Therefore, an optimal substrate/catalyst ratio close to 10 was found by employing 50 mg of catalyst in the reaction medium. This result is an advance compared to previous reports where the optimal substrate/catalyst ratio was ≤5. As is known, a high mass ratio of initial fructose to catalyst is usually preferred in terms of process economics.^[^ ^50^, ^53^ ^]^

#### 2.3.3 Effect of Catalyst Composition (Zr/Sn Ratio)

**Figure 6:**
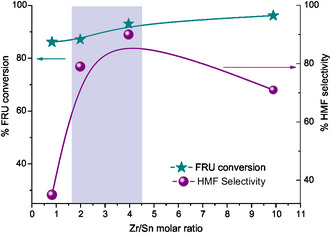
Fructose conversion and HMF selectivity at 150 °C for 120 min in a biphasic system over SO_4_/Sn–Zr–SBA‐15 catalyst with different Zr/Sn molar ratios.

A series of SO_4_/Sn–Zr–SBA‐15 materials with different Zr/Sn molar ratios (from 9.89 to 0.82) were prepared, and additional catalytic experiments were carried out with these catalysts in order to evaluate the optimal relationship of the two metals in the solid for dehydration of fructose to HMF in a biphasic system. The results attained for different Zr/Sn ratio catalysts in terms of fructose conversion and HMF selectivity are presented in **Figure** **6**. In addition, the acid properties of the different Zr/Sn ratio catalysts calculated from FT‐IR measurements with pyridine adsorption and desorption at different temperatures are provided in Table S3, Supporting Information. In general, fructose conversion ranges from 85 to 98%, while selectivity to HMF goes from 27 to 90% approximately. It is noteworthy that employing a Zr/Sn molar ratio of 1.98 in the catalyst, the highest selectivity to HMF is reached, while fructose conversion is the lowest from the series of different Zr/Sn ratio catalysts (see Figure 6). These results indicate that a medium B/L ratio (close to 1) is more adequate to increase HMF selectivity in fructose dehydration. Besides, this indicates that both Bronsted and Lewis acid sites are required in this reaction, as previously discussed in Section 3.3.1.

**Figure 7:**
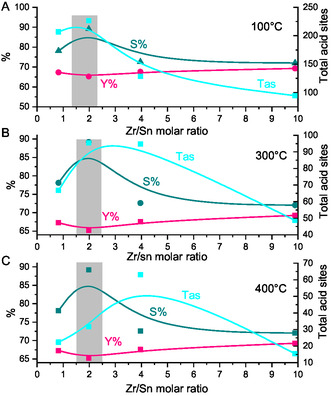
Analysis of total acid sites (Tas), HMF selectivity, and HMF yield as a function of the molar Zr/Sn ratio of SO_4_/Sn–Zr–SBA‐15 materials. Tas measured at different temperatures: A) 100 °C; B) 300 °C; and C) 400 °C.

In order to obtain a better understanding of how the metal composition (Zr/Sn molar ratio) affects the catalytic performance of SO_4_/Sn–Zr–SBA‐15 catalyst, the dependence of total acid sites (Tas), HMF yield (%), and HMF selectivity (%) as a function of increasing Zr/Sn molar ratio is depicted in **Figure** **7**.

In sulfated systems such as SO_4_/Sn–Zr–SBA‐15, sulfate species act as the primary source of protons. These protons are weakly coordinated to the oxygen of the sulfate and can be easily released during the reaction, creating active Brønsted acid sites on the surface. These sites are critical in the dehydration of fructose to HMF, as they promote the protonation reactions necessary for bond cleavage and water elimination. The combined presence of Zr and Sn in a sulfate‐modified catalyst has a synergistic effect on modulating the types of acidity. In catalysts containing both Zr and Sn metals, sulfate groups may interact differently with each metal, allowing for the adjustment of the proportion of Lewis and Brønsted acid sites. Zr, being highly coordinated with sulfate, tends to form more Brønsted sites than Sn. This means that the ratio between Zr and Sn in the catalyst can be utilized to adjust the balance between Brønsted and Lewis sites, maximizing catalytic efficiency.

In this sense, Cara et al.^[^ ^54^ ^]^ investigated the design of mesostructured acid catalysts and demonstrated that the dehydration activity is related to the quantity and strength of Bronsted acid sites. It was also observed that Lewis acid sites experiment deactivation due to water adsorption. Our materials exhibit an adequate catalytic activity with a B/L ratio close to 1 and maximized quantities of total acid sites, as well as lower densities of acid sites.

#### 2.3.4 Study of the Optimized Catalyst (SO_4_/Sn–Zr–SBA‐15, Zr/Sn Ratio =1.98)

Based on the obtained results, the sulfated Sn–Zr–SBA‐15 sample with the best catalytic performance was subjected to scanning electron microscopy (SEM) coupled with energy‐dispersive X‐ray spectroscopy (EDS) and X‐ray photoelectron spectroscopy (XPS) measurements. These analyses were carried out on both fresh and used SO_4_/Sn–Zr–SBA‐15 sample in order to gain a better understanding of the catalytic behavior of this material.

**Figure 8:**
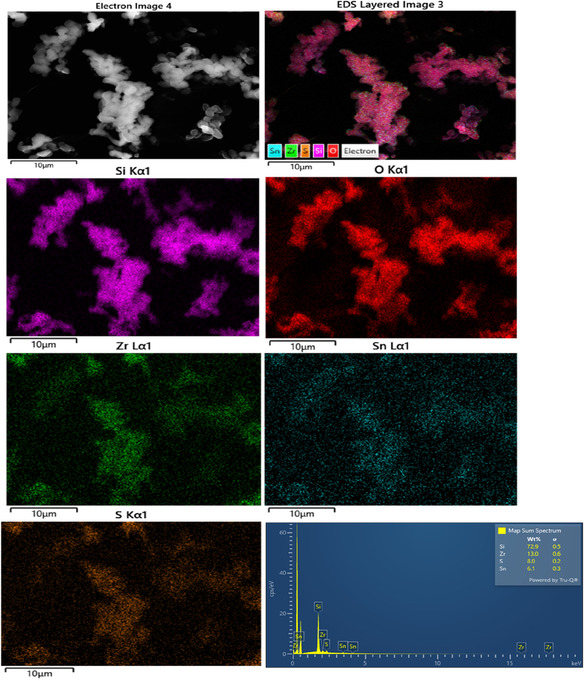
SEM images of SO_4_/Sn–Zr–SBA‐15 with its corresponding EDS compositional analysis.

As shown in SEM–EDS images of **Figure** **8**, the morphology and structure of the SO_4_/Zr–Sn–SBA‐15 mesoporous sample retains the SBA‐15 chain‐like aggregate macrostructure, with no significant impact on its surface or macrostructure after tin impregnation. This indicates that the incorporation of tin does not significantly affect the morphology compared to pure SBA‐15 precursor, which is composed of uniformly sized rope‐like domains.

The EDS spectrum detected signals corresponding to Si, O, Zr, S, and Sn. The resulting Si/Zr and Si/Sn atomic ratios are 18.2 and 63.8, respectively. The Si/Zr ratio is slightly smaller but close to the theoretical, which indicates either a shadowing effect or a local abundance in Zr incorporation on its surface. The sulfur concentration of 8 wt% is consistent with a Si/S molar ratio of 10.4, and it is expected that the majority of sulfate groups are linked to Lewis's acid sites, as it cannot be directly grafted to silanol groups. It is likely that the Sn sites have also been modified by the acidic treatment, serving as anchoring Lewis sites for sulfation, as supported by the NH_3_‐TPD analysis (section 3.3). Our studies agree with those of Marikutsa et al.,^[^ ^47^ ^]^ who studied the effect of tin oxide sulfation by NH_3_‐TPD analysis. They have found that sulfation of tin oxides generates higher surface acidity, and the desorption band of NH_3_‐TPD from sulfated tin species is more clearly defined, demonstrating that the obtained surface acidity is explained by the local inductive effect of electronegative sulfate groups on tin cations.

**Figure 9:**
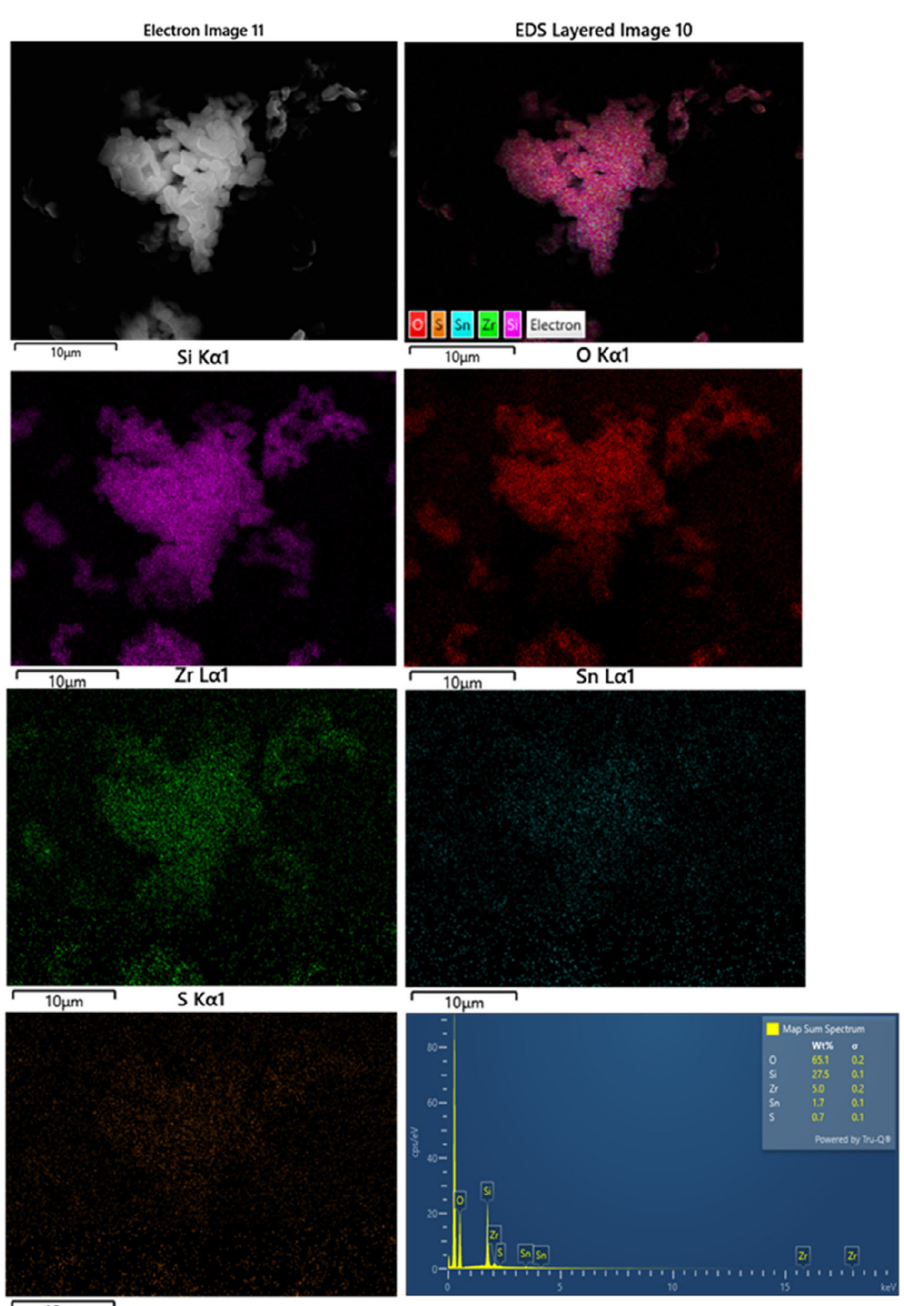
SEM images of SO_4_/Sn–Zr–SBA‐15 used catalyst with its corresponding EDS compositional analysis.

For comparison, SEM images of the SO_4_/Sn–Zr–SBA‐15 used catalyst are shown in **Figure** **9** to confirm the morphology of this material after catalytic tests in the fructose dehydration reaction. As can be observed in Figure 9, the morphology and structure of the mesoporous material remain practically unchanged. Consequently, its use as catalyst in dehydration reactions does not cause appreciable surface modifications. In addition, EDS mapping reveals that the atomic ratios of Si/Zr and Si/Sn were preserved with values of 17.9 and 68.7, respectively. However, a higher Si/S ratio was observed, from 11.2 to 45.0. This confirms the S species leaching in this sulfated material occurring during the fructose dehydration process under reaction conditions, as was previously mentioned (see **Table** **5**).

**Table 5:** Compositional analysis by SEM–EDS microscopy of SO_4_/Sn–Zr–SAB‐15 catalyst before (fresh) and after (used) catalytic reaction.

Material	Molar ratio		
	Si/Zr	Si/Sn	Si/S
SO_4_/Sn–Zr–SBA‐15fresh	16.6	59.9	11.2
SO_4_/Sn–Zr–SBA‐15used	17.9	68.7	45.0

**Figure 10:**
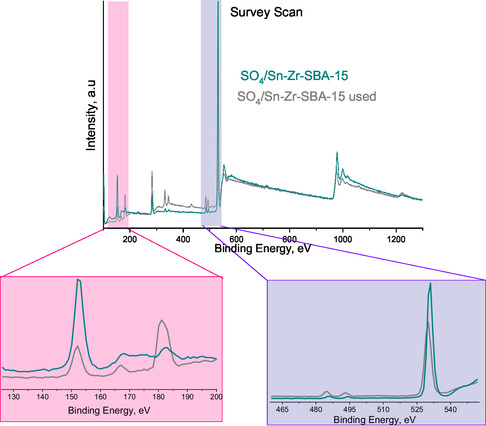
XPS survey scan of SO_4_/Sn–Zr–SBA‐15 sample before (fresh) and after (used) catalytic reaction.

**Figure 11:**
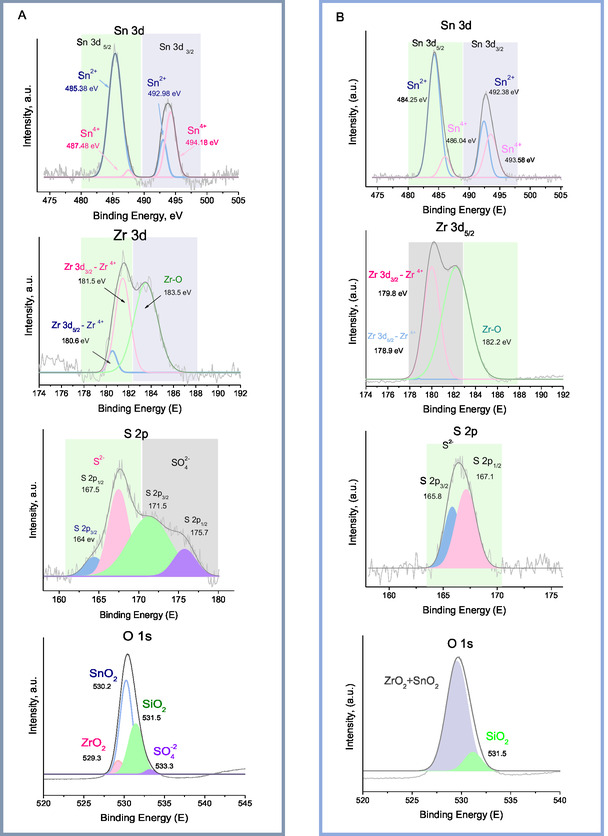
XPS measurements for Sn, Zr, S, and O elements in SO_4_/Sn–Zr–SBA‐15 material: A) fresh catalyst and B) used catalyst.

Additionally, the surface chemical composition of the synthesized SO_4_/Sn–Zr–SBA‐15 sample before (fresh) and after (used) the catalytic reaction was studied using the XPS technique to observe possible changes or modifications with catalyst usage in the fructose dehydration reaction. **Figure** **10** displays wide scan spectra (XPS survey) of the synthesized samples, and no significant changes in the elements under analysis can be observed. **Figure** **11** depicts the Sn 3*d*, Zr 3*d*, S 2*p,* and O 1*s* core level XPS spectra of SO_4_/Sn–Zr–SBA‐15 fresh catalyst. The XPS spectrum of Sn 3*d* shows two symmetric peaks as spin–orbit doublets located at around 485 and 493 eV, which can be assigned to Sn 3*d*5/2 and Sn 3*d*3/2, respectively, confirming the presence of Sn^+4^ ions. In fact, the XPS of Sn 3*d* exhibits results with two clearly distinguished tin oxide (SnOx) components in Sn 3*d* 5/2 (Figure 11). The dominant peak with a large area, located at 487.6 eV, could potentially be associated with Sn(IV) species in which Sn^4+^ replaces Si^4+^ atoms.^[^ ^55^ ^]^ However, the presence of other tin oxide species cannot be excluded. Additionally, the deconvoluted peak at a lower binding energy of (484.7 eV) reveals the presence of different tin oxide species that may be associated with extra‐framework tin oxide nanoparticles.^[^ ^56^ ^]^

Complex shapes were observed for the 3*d* spectral regions of zirconium, which are assigned to the Zr 3*d*5/2 and Zr 3*d*3/2 peak pairs, which have a specific spin‐orbital splitting of 2.1 eV. The successive functionalization and sulfation treatments affect the intensity ratio of both Zr 3*d* peak pairs. The binding energy of Zr 3*d*5/2 is found at 182.3 eV and is assigned to Zr(IV) in the form of ZrO_2_. The formation of Zr(IV) species attached to more electron‐attractive species is found in the energy region of 183.6 eV (Zr 3*d*3/2).^[^ ^57^, ^58^ ^]^ The lower intensity of the Zr 3*d*5/2 peak detected in this material is consistent with the XRD data showing the formation of small clusters of crystalline ZrO_2_. The S 2p spectrum (Figure 11) exhibits four peaks; the peaks at low binding energy at 161.4 and 167.5 eV were associated with S 2*p*3/2 and S 2*p*1/2, respectively, implying a valence of −2, whereas the peaks at 171.5 and 175.7 were assigned to S 2*p*3/2 and S 2*p*1/2, correspondingly, with a predominance of S (VI) species (SO_4_^−2^).^[^ ^59^, ^60^ ^]^

An analysis of the oxygen element (O 1*s*) reveals four peaks. The first (small) peak centered at 529.3 eV is assigned to oxygen atoms in the zirconium structure. Similarly, the second peak centered at 530.2 eV is assigned to oxygen bonded to species of tin. A third peak of O 1*s* centered around 531.5 eV is attributed to oxygen atoms in the silicon lattice, as well as to OH groups, and a final peak centered at 533.3 eV can be attributed to the presence of oxygen in sulfates.^[^ ^61^, ^62^ ^]^ The concentration of chemical groups on the surface of SO_4_/Sn–Zr–SBA‐15 measured by XPS was calculated, revealing a composition of 27.62% Sn^4^^+^ and 42.97% Zr^4^^+^, while the concentration of SO_4_^2^^−^ ions was found to be 59.09%.

Comparative analysis of the XPS results of fresh and used SO_4_/Sn–Zr–SBA‐15 catalysts in Figure 11A,B allowed us to conclude that there are no significant differences for the Zr and Sn species between fresh and used samples, but some differences could be observed mainly in the case of sulfur S 2p. Thus, XPS analysis of sulfur shows that the four initial peaks observed in the SO_4_/Sn–Zr–SBA‐15 fresh sample were reduced to two in the SO_4_/Sn–Zr–SBA‐15 used sample. The peaks (Figure 11B) at low binding energy associated with S 2*p*3/2 and S 2*p*5/2, where sulfur exhibits the oxidation state −2, are still preserved. However, the peaks associated with sulfur in the +6 oxidation state do not appear. This indicates that the loss of sulfur during the reaction mainly occurs in the form of sulfate or S(VI) species. This is consistent with previous inductively coupled plasma ‐ optical emission spectrometry (ICP‐OES), elemental analysis (EA), and SEM–EDS measurements, where a loss or leaching of S species (SO_4_^=^) during the reaction can also be observed.

### 2.4 Reusability of the SO_4_/Sn–Zr–SBA‐15 Catalyst

The possibility of reusing heterogeneous catalysts is another key aspect when evaluating their catalytic performance. In this context, the reuse of SO_4_/Zr–Sn–SBAS‐15 material in the fructose dehydration was evaluated at optimal conditions, at 150 °C for 120 min of reaction, by employing the same catalyst in successive catalytic cycles, without any regeneration treatment. Thus, the used catalyst was recovered from the reaction mixture by filtration, rinsed with deionized water, and then dried in an oven at 80 °C overnight. As mentioned, no further thermal or chemical regeneration procedures were followed.

**Figure 12:**
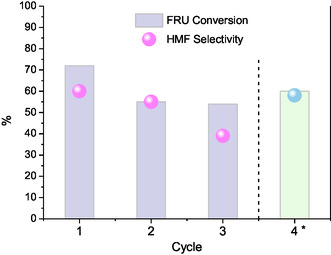
Recycling tests of SO_4_/Sn–Zr–SBA‐15 catalyst at 150 °C for 120 min, without regeneration (cycles 1, 2, and 3), and with regeneration step (cycle 4*).

As shown in **Figure** **12**, fructose conversion decreased from the first use to the second one and then to the third use along three consecutive recycles of the catalyst. Thus, the first use resulted in a fructose conversion of 73.1%, while it decreased to 56% and 51% for the second and third uses, respectively. The selectivity toward HMF also suffered a decrease from 65.2% to ≈40% after three catalytic cycles, indicating that further regeneration of active sites is required for this material in the mentioned reaction conditions. The decrease in conversion and selectivity observed over multiple cycles would indicate a pore blocking by the deposition on the solid catalyst of undesirable organic products (humins), which was evidenced by the change of color of the catalyst before (light yellow) and after (dark brown or black) the reaction.

In view of these results, another recycling test was performed with the SO_4_/Sn–Zr–SBA‐15 catalyst recovered after the first catalytic cycle by filtration, rinsed with deionized water, and dried overnight at 80 °C and then regenerated by calcination in air at 500 °C for 5 h before being tested in a new catalytic reaction. The results attained in this catalytic reuse test with the regenerated catalyst are denoted as Cycle 4* in Figure 12.

As can be seen (Figure 12), the calcination procedure mostly regenerates the active sites of the catalyst, releasing most of the carbonaceous deposits from the pores of the solid and partially restoring its activity. Thus, fructose conversion increases with respect to the previous second and third cycles, while the HMF selectivity is maintained at practically similar values when compared with the fresh catalyst.

The removal of deposited carbonaceous compounds from the pores and over the surface of the catalyst after calcination treatments was evidenced by elemental analysis (EA) of the fresh and regenerated catalysts (see **Table** **6**). Additionally, ICP analysis of fresh and regenerated catalysts shows that although there is no loss of Sn and Zr in the reaction medium, a small loss of S species (sulfate groups) to the reaction medium could be detected (Table 6). This fact could explain the decrease in catalytic activity due to the decrease in the amounts of sulfate groups present in the catalyst, this reducing the number of active sites available in the mesoporous solid.^[^ ^51^ ^]^

**Table 6:** EA and ICP analysis of SO_4_/Sn–Zr–SBA‐15 catalyst before (fresh) and after (used) catalytic reaction.

Catalyst	Sn [wt%]	Zr [wt%]	S [wt%]	C [wt%]	N [wt%]	H [wt%]
Fresh catalyst	1.72	4.06	2.67	0.13	0.00	1.29
Regenerated catalyst	1.83	4.60	0.34	0.08	0.00	0.54

In any case, the reported activity of the optimized SO_4_/Sn–Zr–SBA‐15 catalyst in fructose dehydration, performed at 150 °C for 120 min of reaction, was higher than the activity obtained with the analogous Zr–Sn‐containing mesoporous materials without any sulfation procedure (see Figure 4). This procedure together with the adequate way and order of Zr and Sn metal incorporation are key factors to achieve a better performance of mesoporous solids in fructose dehydration in a biphasic system.

## 3 Conclusions

Catalysts based on zirconium (Zr) and tin (Sn) incorporated into the SBA‐15 material, which also contains sulfate groups, have been prepared and characterized using various techniques. XRD and SEM–EDS measurements confirmed that the mesoporous structure and morphology of SBA‐15 were preserved after different postsynthesis treatments and even after consecutive catalytic cycles in the selective fructose dehydration to HMF. Moreover, FTIR analysis of adsorbed pyridine revealed the presence of both Lewis and Brønsted acid sites in all samples. It was observed that the relative strength of Brønsted and Lewis acid sites depends on the postsynthesis procedure, which affects the sulfonation of Zr and Sn sites.

The SO_4_/Sn–Zr–SBA‐15‐type materials, containing sulfated zirconia and tin, were more active in the dehydration of fructose to HMF compared to analogous systems containing only one of the metals or those that were not subjected to the postsynthesis sulfation procedure. The main reaction parameters were investigated using the best‐performing SO_4_/Sn–Zr–SBA‐15 catalyst. The highest fructose conversion (≈90%) and HMF selectivity (>83%) values were achieved by working at 150 °C during 120 min in an aqueous/organic biphasic system with an organic solvent mixture MIBK : 2‐butanol (7:3) and with a high initial concentration of fructose (30 wt%), which contrasts with much lower concentrations commonly reported in previous studies. This catalytic behavior was influenced by the metal dispersion and the adequate strength of Brønsted and Lewis active sites. The most active SO_4_/Sn–Zr–SBA‐15 catalyst exhibited a higher ratio of Bronsted/Lewis (B//L) acid sites of moderate strength compared to the other catalytic samples evaluated, primarily due to the presence of well‐dispersed tin and zirconium species, as well as to the enhanced acidic properties of the sulfated groups present in this material. Additionally, the study of the Zr/Sn molar ratio in this solid catalyst on the fructose‐to‐HMF dehydration reaction demonstrated that the Zr/Sn molar ratio of 1.98 in the SO_4_/Sn–Zr–SBA‐15 material was optimal in terms of fructose conversion and selectivity to the desired HMF product.

The stability of the SO_4_/Sn–Zr–SBA‐15 material was examined over consecutive catalytic cycles under optimal reaction conditions. Data indicated that after three consecutive cycles, the catalytic activity toward the fructose‐to‐HMF dehydration (in terms of fructose conversion) partially decreased when no regeneration procedures are implemented. Thermal regeneration treatment effectively removed undesirable carbonaceous deposits from the surface and pores of the mesoporous material, as confirmed by EA measurements, and partially restored the initial catalytic activity. Comparative characterization studies of the catalyst before (fresh) and after (used) the catalytic reaction allowed us to conclude that, despite the negligible loss of Zr and Sn species detected by ICP measurements, the leaching of SO_4_^=^ species into the reaction medium may be the primary factor responsible for the decrease in catalytic activity under reaction conditions. Further studies to enhance the stability of this Zr–Sn‐based mesoporous solid, which is emerging as a promising and viable candidate for future research in saccharide dehydration reactions, will focus on the stabilization of Zr and Sn on the mesoporous framework, combined with low‐cost‐adapted sulfonation procedures (i.e., grafting of S species), among others.

## 4 Experimental Section

4.1

4.1.1

##### Synthesis of Acid Materials

The SBA‐15 mesoporous material was synthesized using the sol–gel technique^[^ ^19^ ^]^ by preparing an acidic solution (H_2_O + HCl), in which the structure‐directing agent (P123) was dissolved. This solution was maintained under agitation at 50 °C. Subsequently, the silicon source (TEOS) was added dropwise under vigorous stirring for 5 min, and the mixture was kept stationary for 20 h. The mixture was then placed into a closed polypropylene (PP) vessel and heated in an oven at 80 °C for 48 h. After cooling, the solid was filtered, washed, and dried overnight in the oven. The molar ratio used for the synthesis gel was 1 TEOS: 158 H_2_O: 6 HCl: 0.016 P123. To remove the template, a nonoxidative calcination was carried out under a nitrogen flow (20 mL min^−^^1^), with the temperature ramped at 2 °C min^−^^1^ up to 500 °C and held at that temperature for 5 h. In a second step, the material was calcined under an oxygen atmosphere using the same temperature program. The resulting material was designated as **SBA‐15**.

The incorporation of metals and the sulfation of the SBA‐15 mesoporous silica matrix were performed via two‐ or three‐step procedure, as described below.

Zirconium incorporation on SBA‐15: Zirconia‐functionalized SBA‐15 was synthesized by wet impregnation using hydrated zirconium oxychloride (ZrOCl_2_·xH_2_O) as the Zr source. This precursor was dissolved in water under stirring, and the SBA‐15 support was added in sufficient quantity to obtain Si/Zr molar ratios of 10, 20, and 30 in the final solid. The mixture was then subjected to rotary evaporation until dry. The resulting solid was further dried in an oven overnight at 60 °C and calcined an oxygen atmosphere at 500 °C for 5 h, using a heating rate of 3 °C min^−^^1^. This sample was designed as Zr–SBA‐15.

Coimpregnation of tin and zirconium on SBA‐15: A subsequent treatment involved the simultaneous coimpregnation of zirconium and tin onto SBA‐15. The procedure was similar to that used for the Zr–SBA‐15 synthesis. In this case, 2, 5, and 8 wt% of the tin source [Sn(CH_3_)_2_Cl_2_] were added along with the zirconium source to an aqueous solution containing the support, maintaining Si/Zr molar ratios (10, 20, and 30). The synthesis followed the same steps as for Zr–SBA‐15, yielding the sample **Sn–Zr–SBA‐15**.

Sulfation procedure: The metal‐modified SBA‐15 materials were further treated to incorporate SO_4_^2^^−^ species. Sulfation was conducted by suspending the corresponding material in a 10 vol% aqueous H_2_SO_4_ solution and stirring for 12 h. The solid was then filtered, dried in an oven at 100 °C for 3 h, and calcined under an air atmosphere at 500 °C for 5 h. The sulfation procedure was applied either as a final modification or between the Sn and/or Zr incorporation steps, depending on the synthesis route.

By alternating these steps, the following samples were obtained: 1) **SO**_**4**_ **/Zr–SBA‐15:** SBA‐15 functionalized with sulfated zirconia. 2) **Sn–SO**_**4**_ **/Zr–SBA‐15:** SBA‐15 impregnated with zirconia, then sulfated, and subsequently impregnated with tin. 3) **SO**_**4**_ **/Sn–Zr–SBA‐15:** SBA‐15 coimpregnated with zirconia and tin, followed by sulfation

##### Catalyst Characterization

The X‐ray diffractograms were collected using an X’Pert Pro PANalytical diffractometer with a CuKα radiation source (*λ* = 1.5418 Å). The measurements were performed over a 2*θ* range of 0.3–8° for small‐angle diffraction and 20–120° for wide angle, with a scanning rate of 0.02° and an accumulation time of 20 s per step.

Fourier transform infrared spectroscopy (FTIR) spectra were acquired using a Nicolet 5700 spectrometer equipped with a Harris Praying Mantis diffuse reflectance accessory, an environmental chamber for in situ measurements, and a high‐sensitivity MCT‐A detector. Prior to analysis, the catalysts were dried at 120 °C for 2 h under a helium flow, exposed to pyridine vapor, and then degassed with helium for 30 min to remove physiosorbed pyridine. The samples were then outgassed at different temperatures (room temperature (RT), 150, and 200 °C) and cooled before spectra acquisition.

NH_3_‐TPD analyses were conducted using a Micromeritics Autochem II equipped with a thermal conductivity detector. Prior to analysis, the catalyst samples were dried and degassed at 100 °C for 2 h, followed by NH_3_ adsorption for 1 h. Physiosorbed ammonia was removed by flowing helium at 100 °C for an additional hour. TPD was then conducted by heating the samples at 10 °C min^−^^1^ up to 700 °C under a helium flow.

N_2_ adsorption–desorption isotherms were measured at −196 °C using an ASAP 2020 system after degassing the samples at 400 °C. Pore size distribution was calculated using nonlocal density functional theory applied to the adsorption branch, assuming cylindrical pore geometry.

SEM images were obtained using an FE–SEM Σigma high‐resolution microscope operating at 20 kV, equipped with a Schottky‐type field emission electron gun (FEG) and both secondary and a backscattered electron detector. XPS measurements were carried out using a Thermo Scientific K‐Alpha instrument with a monochromatic Al Kα source (hv = 1486.6 eV, 150 W). The base pressure in the analysis chamber was 10^−^^8^ Pa, and an internal standard C 1*s* at 284.8 eV was used to correct the charge change. High‐resolution spectra of the elements O, Si, S, Sn, and Zr were acquired with pass energy 25 and 10 eV, step size 1 and 0.1 eV, respectively. The analyses were recorded at RT with a Phoibos150‐MCD.

##### Catalytic Evaluation

Catalyst tests for the selective dehydration of fructose to HMF were carried out using a glass tube reactor (15 mL nominal volume, 10 bar maximum pressure; Ace Pressure Tube, Sigma‐Aldrich) equipped with magnetic stirring. For each reaction, 1.5 mL of 30 wt% fructose aqueous solution (FA), 3.5 mL of an organic phase as an extracting agent (FO), and 50 mg of catalyst were introduced into the reactor. The organic phase consisted of MIBK: 2‐butanol in a 7:3 weight ratio. The reactor was immersed in a preheated and stirred silicone oil bath, and zero time was established at the immersion time for better reproducibility.

The main reaction parameters such as reaction times (20, 30, 60, 90, 120, 150 min), temperatures (120, 130, 140, 150 °C), and catalyst loadings (25, 50, 75 mg) were varied to identify the optimal conditions for maximizing HMF yield. The influence of different Zr/Sn ratios in the catalyst on the fructose conversion and the selectivity to HMF under optimal reaction conditions was also evaluated by employing SO_4_/Sn–Zr–SBA‐15 materials with Zr/Sn ratios of 0.98, 1.98, 3.96, and 9.89. Additionally, the stability and durability of the catalyst were evaluated during several catalytic cycles under optimal conditions. After the reaction, the solid catalyst was recovered from the reaction mixture by filtration and rinsed with abundant deionized water, and then it was dried in air at 80 °C overnight. Finally, further thermal calcination treatment (regeneration) was followed to regenerate the catalyst.

The analysis of the reaction products was performed using a high pressure liquid chromatography Jasco UV‐975/PU‐980 equipped with refractive index (RI) and UV–visible detectors. The products were separated in an Aminex HPX‐87H column, using H_2_SO_4_ 5 mM solution as the mobile phase at 50 °C with a 0.6 mL min^−1^ flow. The quantification of products was carried out using calibration curves with pure standards. Additionally, in selected experiments, the reaction products in the organic phase were analyzed by gas chromatography employing a Petrocol DH 100 capillary column coupled with an FID detector.

## Conflict of Interest

The authors declare no conflict of interest.

## Supporting information

Supplementary Material

## Data Availability

The data that support the findings of this study are available from the corresponding author upon reasonable request.
