# Supplementary material for: Synthesis of Sulfated Sn–Zr Mesoporous Catalysts for the Selective Dehydration of Hexose‐Type Monosaccharides
Source: ChemistryOpen. 2025 Jul 13;14(10):e202400480. doi: 10.1002/open.202400480 (PMC12518035; doi:10.1002/open.202400480)
Supplement: Supplementary file 1 — Supplementary Material [file OPEN-14-e202400480-s001.pdf]

SUPPORTING INFORMATION

SYNTHESIS OF SULFATED-Sn-Zr-MESOPOROUS CATALYSTS FOR THE SELECTIVE DEHYDRATION OF HEXOSE-TYPE MONOSACCHARIDES

Eliana Diguilio[1], Horacio Falcón[1], Marcelo E. Domine[2], María Laura Martínez[3]**

[1] *Centro de Investigación y Tecnología Química (CITeQ-CONICET), Universidad Tecnológica Nacional - Facultad Regional Córdoba, Maestro M. López esq. Cruz Roja, 5016 Córdoba, Argentina*

[2] *Instituto de Tecnología Química (UPV - CSIC), Universitat Politècnica de València. Consejo Superior de Investigaciones Científicas, Avda. Los Naranjos s/n, 46022, Valencia, España*

[3] *Centro de Investigación en Nanociencia y Nanotecnología (NANOTEC), Universidad Tecnológica Nacional - Facultad Regional Córdoba, Maestro M. López esq. Cruz Roja, 5016 Córdoba, Argentina*

**mdomine@itq.upv.es; mmartinez@frc.utn.edu.ar*

Table of Contents

Figure S1: Variation of surface area, average pore size and pore volume of SBA-15 support after successive treatments (wet impregnation and sulfation)

Figure S2: Fructose conversion and HMF yield at different temperatures (a); at different reaction times (b); with different amount of SO_4/Sn-Zr-SBA-15 catalyst, employing fructose aqueous solution (30wt.%) and solvent mixture MIBK-2:butanol (7:3).

Table S1: Brønsted and Lewis acid sites of Zr-SBA-15 and Sn-Zr-SBA-15 determined by FTIR of pyridine adsorption and ulterior desorption at 100, 200, 300 and 400 °C.

Table S2: Brønsted and Lewis acid sites of SO_4/Zr-SBA-15, Sn-SO_4/Zr-SBA-15 and SO_4/Sn-Zr-SBA-15 determined by FTIR of pyridine adsorption and ulterior desorption at 50, 100 and 200 °C.

Table S3. Bronsted and Lewis acid sites of SO_4/Sn-Zr/-SBA-15 catalyst with different Zr/Sn mass ratio determined by FTIR of pyridine desorbed at different temperatures (100, 300 and 400°C).

Figure S3: Bronsted and Lewis acid sites concentrations determined by FTIR measurements with Py desorption at 200 ºC for different SBA-15-based materials used in this study

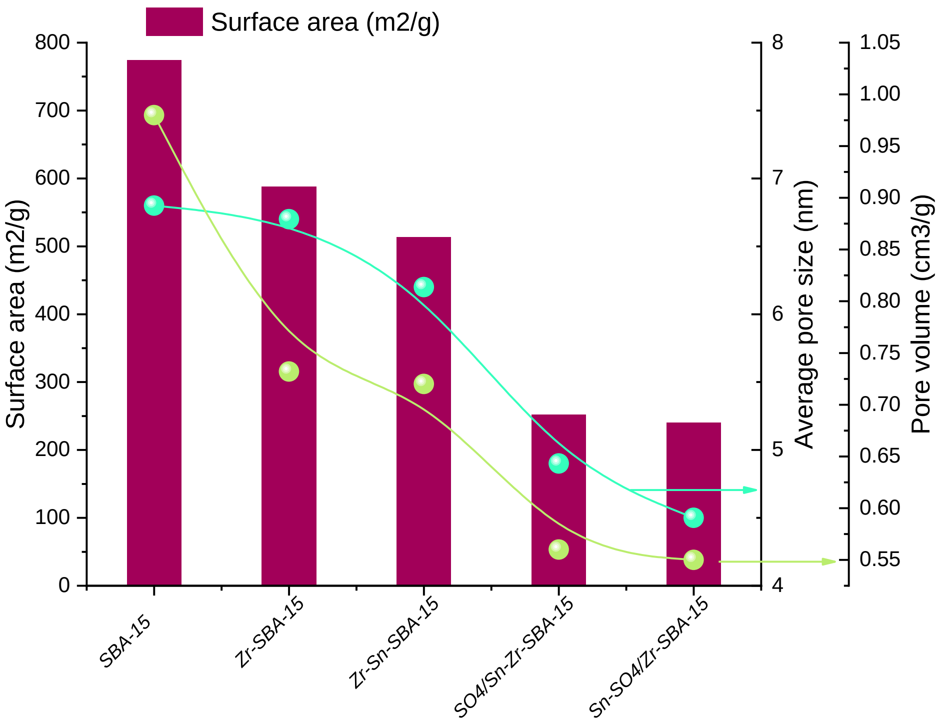

Figure S1: Variation of surface area, average pore size and pore volume of SBA-15 support after successive treatments (wet impregnation and sulfation)

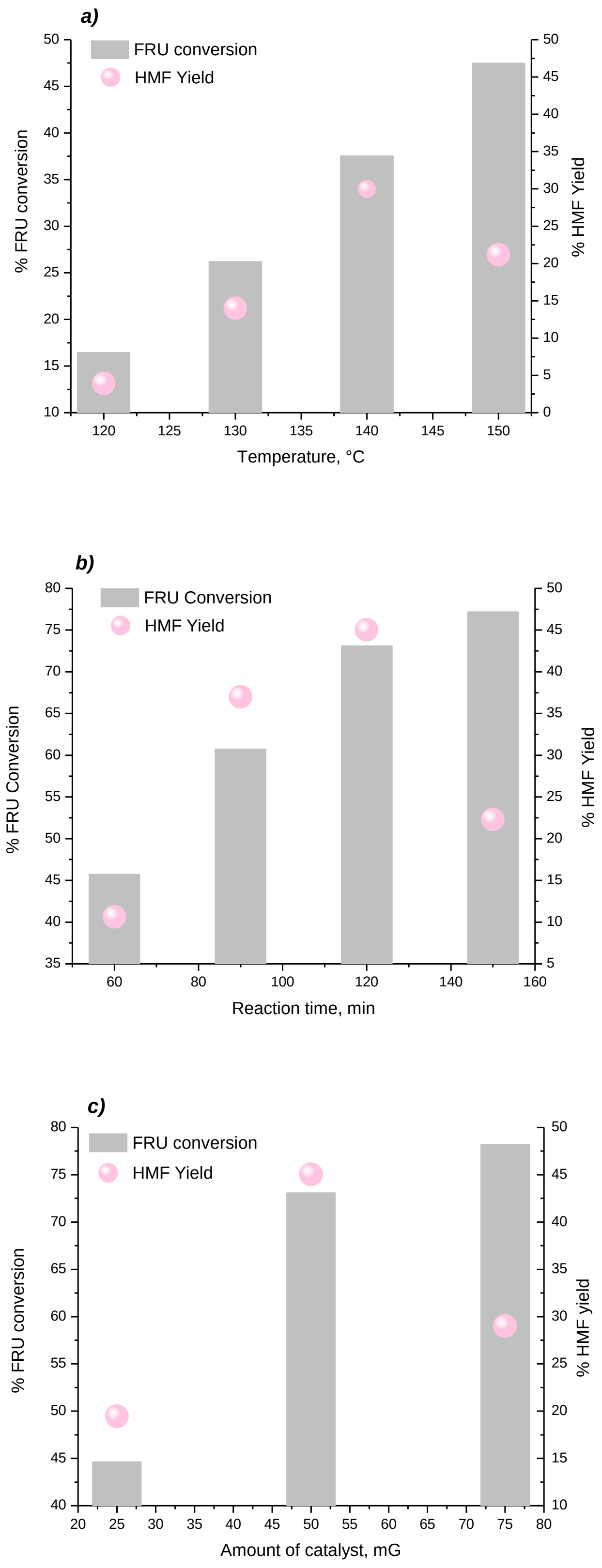

Figure S2: Fructose conversion and HMF yield at different temperatures (a); at different reaction times (b); with different amount of SO_4/Sn-Zr-SBA-15 catalyst, employing fructose aqueous solution (30wt.%) and solvent mixture MIBK-2:butanol (7:3).

Table S1: Brønsted and Lewis acid sites of Zr-SBA-15 and Sn-Zr-SBA-15 determined by FTIR of pyridine adsorption and ulterior desorption at 100, 200, 300 and 400 °C.

Catalyst: Zr-SBA-15

T(°C)	L(μmol Py/gr)	B(μmol Py/gr)	B/L	Total acid sites
100	118.92	41.33	0.34	160.26
200	48.72	28.45	0.58	77.17
300	36.09	15.64	0.43	51.74
400	20.34	9.22	0.45	29.56

Catalyst: Sn-Zr-SBA-15

T(°C)	L(μmol Py/gr)	B(μmol Py/gr)	B/L	Total acid sites
100	142.78	50.96	0.35	193.74
200	56.02	28.65	0.51	84.68
300	48.10	18.68	0.38	66.78
400	28.79	10.43	0.36	39.22

Table S2: Brønsted and Lewis acid sites of SO_4/Zr-SBA-15, Sn-SO_4/Zr-SBA-15 and SO_4/Sn-Zr-SBA-15 determined by FTIR of pyridine adsorption and ulterior desorption at 50, 100 and 200 °C.

Catalyst: SO_4/Zr-SBA-15

T(°C)	L (μmol Py/gr)	B (μmol Py/gr)	B/L ratio	Total acid sites
50	805.32	83.31	0.10	888.64
100	409.03	95.87	0.23	504.91
200	187.23	79.47	0.42	266.71

Catalyst: Sn-SO_4/Zr-SBA-15

T(°C)	L (μmol Py/gr)	B (μmol Py/gr)	B/L ratio	Total acid sites
50	265.62	106.97	0.40	372.59
100	69.02	79.30	1.14	148.32
200	49.40	45.90	0.92	95.31

Catalyst: SO_4/Sn-Zr-SBA-15

T(°C)	L (μmol Py/gr)	B (μmol Py/gr)	B/L ratio	Total acid sites
50	553.47	286.07	0,.51	839.54
100	254.93	482.13	1.89	737.06
200	125.64	420.44	3.34	546.08

Table S3. Bronsted and Lewis acid sites of SO_4/Sn-Zr/-SBA-15 catalyst with different Zr/Sn mass ratio determined by FTIR of pyridine desorbed at different temperatures (100, 300 and 400 °C).

T= 100 °C

(Si/Zr)/(Si/Sn) ratios in catalysts	Zr/Sn Molar Ratio	L (μmol Py/gr)	B (μmol Py/gr)	B/L ratio
10/2	9.89	40.53	54.02	1.33
10/5	3.96	17.63	110.88	6.28
20/5	1.98	88.94	137.46	1.54
30/8	0.82	61.43	145.45	2.36

T= 300 °C

(Si/Zr)/(Si/Sn) ratios in catalysts	Zr/Sn Molar Ratio	L (μmol Py/gr)	B (μmol Py/gr)	B/L ratio
10/2	9.89	14,59	34.05	2.33
10/5	3.96	26.23	68.47	2.61
20/5	1.98	40.15	55.26	1.37
30/8	0.82	21.57	45.17	2.09

T= 400 °C

(Si/Zr)/(Si/Sn) ratios in catalysts	Zr/Sn Molar Ratio	L (μmol Py/gr)	B (μmol Py/gr)	B/L ratio
10/2	9.89	5.82	9.61	1.65
10/5	3.96	22.52	40.51	1.80
20/5	1.98	15.27	16.47	1.08
30/8	0.82	6.71	15.63	2.33

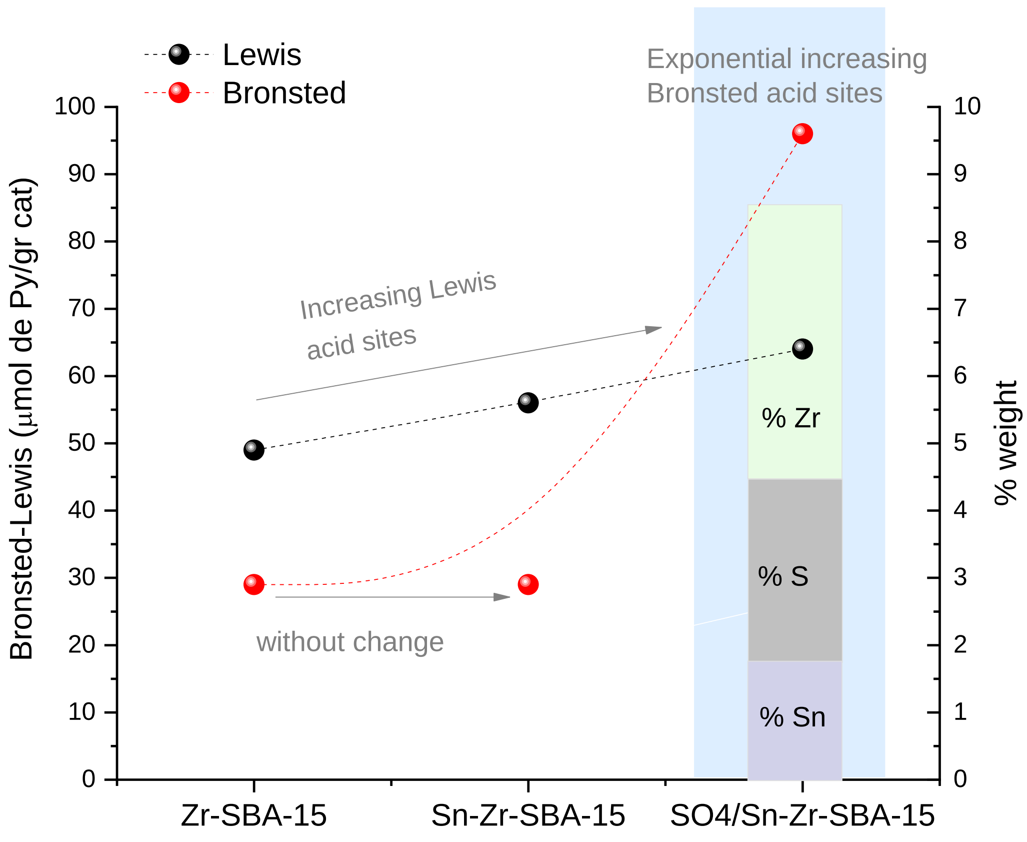

Figure S3: Bronsted and Lewis acid sites concentrations determined by FTIR measurements with Py desorption at 200 ºC for different SBA-15-based materials used in this study.
